# A novel and validated nomogram to predict overall survival for gastric neuroendocrine neoplasms

**DOI:** 10.7150/jca.35785

**Published:** 2019-10-15

**Authors:** Sheng Zhang, Yi Xin Tong, Xin Hua Zhang, Yu Jie Zhang, Xiang Shang Xu, Ai Tang Xiao, Teng Fei Chao, Jian Ping Gong

**Affiliations:** Department of Gastrointestinal Surgery Tongji Hospital, Tongji Medical College, Huazhong University of Science and Technology Jie Fang Ave, No. 1095 Wuhan, China.

**Keywords:** Gastric neuroendocrine neoplasm, Nomogram, Overall survival

## Abstract

Background: This study aims to develop and validate an effective nomogram to estimate the individual outcome of patients with Gastric neuroendocrine neoplasms (G-NENs).

Methods: A total of 260 patients diagnosed with G-NENs at two medical centers were included, with 156 patients allocated as training set and 104 patients as validation. Predictive nomogram was constructed based on multivariate analyses using RMS package in R version. The predictive accuracy and discriminative ability were analyzed by C-index, risk group stratification and calibration curve, which was compared with other predictive systems for G-NENs.

Results: In multivariate analysis, age, Ki-67, mitoses, neutrophil to lymphocyte ratio, serum tumor marker and distant metastasis were significantly associated with overall survival. The constructed prognostic nomogram demonstrated a good calibration and discrimination value with 0.884 and 0.852 C-indices in training and validation dataset. Compare to World Health Organization (WHO) grading system (C-indices=0.760 and 0.732) and American Joint Committee on Cancer (AJCC) tumor-node-metastasis (TNM) staging system (C-indices=0.747 and 0.811), the nomogram displayed a better predictive accuracy.

Conclusions: The novel prognostic nomogram showed superior predictive value in overall survival of G-NENs. It might be a useful tool for clinicians in estimating individual survival in G-NENs patients.

## Introduction

Gastric neuroendocrine neoplasms (G-NENs) are common type of gastroenteropancreatic neuroendocrine neoplasms (GEP-NENs), accounting for about 20% of all neuroendocrine neoplasms (NENs). 1 Although G-NENs are relatively rare malignancies, the incidence of neuroendocrine tumors has increased recently. 1 Compare to gastric cancer, G-NENs have better prognosis but the patients will still suffer from recurrence and metastasis. 2,3 Consequently, it is both necessary and meaningful to explore an effective model to precisely evaluate overall survival of G-NEN patients in clinical practice.

Diversifications in biological behavior and clinical manifestation are predominant features of G-NENs. Thus, to clinically estimate the individual outcome of the G-NEN patients is very difficult. The grading system from World Health Organization (WHO)/European Neuroendocrine Tumor Society (ENETS) and the TNM staging system from the American Joint Committee on Cancer/Union for International Cancer Control (AJCC/UICC) are in use to evaluate outcomes of G-NEN patients. 4-8 Studies have demonstrated that G grading and TNM staging are independent prognostic factors for survival and effective to predict outcomes of G-NEN patients. 9-11 However, while these systems stratify patients into different prognostic groups, they fail to predict individual survival of G-NENs. Therefore, there is essential to develop a predictive model including more prognostic factors to estimate overall survival of G-NENs patients.

Nomogram is a calculating score combining clinical or laboratory parameters to predict individual outcome. 12-14 Many nomograms have been developed to predict outcomes in various types of malignancies. 15-18 Limited numbers of nomograms predicting lymph node metastasis of appendix NET or overall survival of pancreas and small intestine NETs have been reported. 19-21 However, these models included very few prognostic factors. Recently, Fang et al analyzed 1183 GEP-NEN patients and constructed a comprehensive nomogram to predict overall survival. 22 Fang's nomogram included both pancreatic and gastrointestinal NETs, which might not be specific for clinical use. Therefore, we performed a multicenter retrospective analysis aiming to construct (training cohort) and validate (validation cohort) a novel survival prediction model to estimate prognosis of G-NENs patients. In addition, we also compared the predictive accuracy and discrimination ability of this model with current G grading, TNM staging system and Fang's nomogram mentioned above.

## Materials and Methods

### Study design and participants

A multicenter retrospective study was conducted involving two cohorts: a training cohort (score construction and internal validation) and an external validation cohort. Approvals for data collection and patient informed consent were obtained from Institutional Review Board (IRB) in two individual institutions.

For training cohort, we retrospectively collected data of 156 patients who were diagnosed with G-NENs between January 2000 and December 2010 at department of gastrointestinal-pancreatic surgery, 1^st^ affiliated hospital of Sun Yat-Sen University (SYSU). Pathological evidence of G-NENs were achieved from either curative/palliative operation or biopsy. Eligible patients were included based on the following criteria: histopathological confirmed diagnosis of G-NENs (site of the origin was classified as stomach including cardia, body, lesser or greater curvature, angle and antrum). The exclusion criteria were patients with non-gastric NETs, simultaneously with other malignant neoplasm, tumor of uncertain origin or patients with missing key data either in terms of clinical parameters or follow-up reports. The following information were retrospectively collected: 1. Demographic characteristics such as age, gender, primary location; 2. Clinical or laboratory characteristics such as neutrophil to lymphocyte ratio, serum tumor markers as carcinoembryonic antigen (CEA) and carbohydrate antigen 19-9 (CA19-9), tumor size, invasion depth (T), presence of lymph node metastases (N), presence of distant metastases (M), treatment; 3. Pathology characteristics such as ki-67 index, mitotic count, G grade were collected for identification of prognostic factors and construction of the nomogram. G grade was defined according to WHO 2010 classification system. 7 Low grade (G1) is defined as <2 mitoses/10 high power field (HPF) and/or <3% Ki-67 index; Intermediate grade (G2) is defined as 2-20 mitoses/10 HPF and/or 3-20% Ki-67 index; High grade (G3) is defined as >20 mitoses/10 HPF and/or >20% Ki-67 index. We performed telephone interviews or outpatient follow-ups every year. The follow-up period was measured from the time of diagnosis to the date of last follow-up (December 31^st^, 2012) or death date. Overall survival (OS) is the primary endpoint measured from the time of confirmed diagnosis to the last follow-up date (or date of death).

For external validation cohort, we retrospectively collected information of 104 G-NEN patients who met the inclusion criteria at department of gastrointestinal surgery and department of oncology, Tongji Hospital of Huazhong University of Science and Technology (TJH) from April 2011 to December 2016. The same baseline clinical characteristics were collected and patients without data in any of the items in the constructed nomogram were excluded. The follow-up schedules and primary endpoints (OS) were the same as in the training cohort. The last follow-up date of validation cohort is August 31^st^, 2018.

### Assessment of NLR and serum tumor markers

The data of preoperative blood cell counts were collected from the electronic patient record systems. The NLR was calculated as the ratio of absolute neutrophil count divided by the lymphocyte count. Receiver operating characteristic (ROC) curve was performed to determine the optimal cut-off value of NLR. The value of serum tumor marker CEA and CA19-9 was also retrieved from electronic record system, the normal range of CEA and CA19-19 is 0-5μg/L and 0-34μg/L.

### Development and validation of the predictive model

The predictive model was formulated based on the results of the multivariate Cox regression analysis using R language (version 3.1.1, http://www.r-project.org) via the design and survival packages: survival, mice, and Hmisc. 23 Variables included in the predictive model were identified by a backward stepwise selection process with the Akaike information criteria. 24

The performance of the predictive model was evaluated by measuring both discrimination and calibration using bootstraps with 1000 resamples in both training (internal validation) and validation (external validation) cohorts. Discrimination was evaluated with concordance index (C-index) which referred to the model's ability to accurately predict outcomes. The maximum C-index value is 1.0 which indicates that a perfect prediction ability of the nomogram whereas the value 0.5 indicates that it is a random chance to correctly predict the outcome of the nomogram. The larger the value of C-index was, the more accurate the model predicted. Calibration curves were applied to compare the predicted survival and the actual survival. 25 For external validation of the nomogram, the total point of each patient was computed and used for Cox regression. C-index and calibration curves were also applied to evaluate the prognostic efficiency. Additionally, we used the decision curve analysis (DCA) and area under ROC curve (AUC) method to estimate the predictive precision of nomogram. 26

### Risk stratification and quality assessment

According to individual points calculated by the nomogram in both training and validation cohort, we divided patients into 3 risk groups (<25%, 25-75% and >75% percentile quartiles). We accessed the discrimination ability of the nomogram and applied the Transparent Reporting of a multivariable prediction model for Individual Prognosis or Diagnosis (TRIPOD) guideline for our nomogram. 27 Every applicable item in the checklist of TRIPOD guideline was described and reported in this study (Supplemental Table S1).

### Statistical analyses

Continuous variables were presented as medians (range) and analyzed with Student's t-test or Mann-Whitney U test. Categorical variables were reported as whole numbers and percentages. The Kaplan-Meier method was used to evaluate potential prognostic factors. In the univariate statistical analysis, factors associated with outcome was assessed by univariate logistic analysis for quantitative variables and χ2 test for qualitative variables. Only factors with p-value<0.1 in univariate analysis were included in the final multivariate analysis model. Multivariate Cox regression was employed to identify independent prognostic factors for OS and predictive model was formulated based on the results. We only reported variables included in the final model with hazard ratio (HR) and 95% confidence interval (CI). All statistical tests were performed in SPSS version 21 (IBM, Armonk, NY, USA) with a significance level of p-value<0.05.

## Results

### Patient baseline characteristics

**Figure 1:**
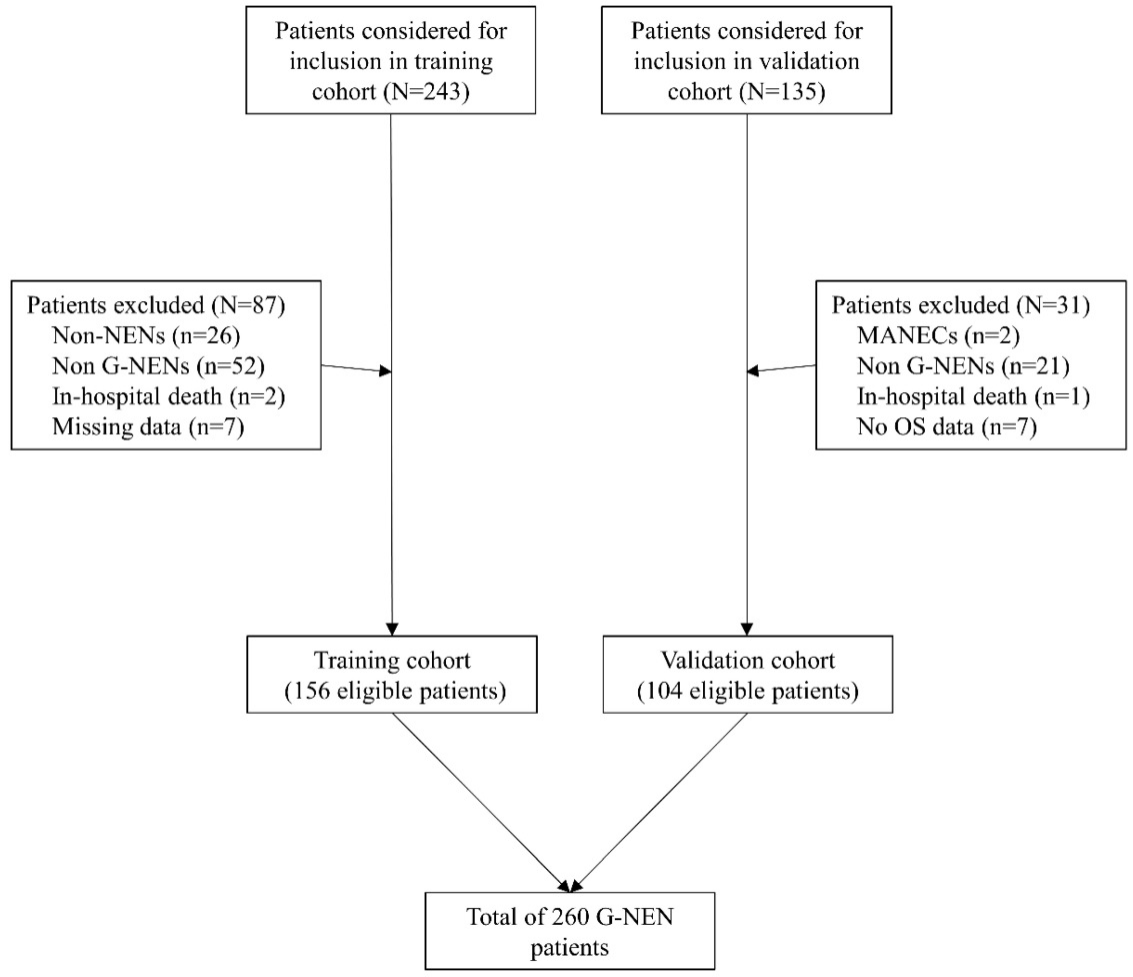
Brief flow chart for patients included in the training and validation cohorts

A total 378 patients were identified and considered eligible for this study. After screening based on inclusion and exclusion criteria, 260 patients were included in final analysis, with 156 patients in the training cohort and 104 patients in the validation cohort. (Figure 1). Most patients were men (170/260, 68%) and the average age is 57±13.1 years old. The distribution of grade for G1, G2, G3 was 32.7% (85/260), 17.3% (45/260) and 50.0% (130/260) respectively. Lymph node metastasis and distal metastasis presented in 66.9% (174/260) and 17.7% (46/260) patients respectively. Curative operation was performed on 78.1% (203/260) patients. Palliative operation was defined as R1/R2 resection of the primary tumor site or patients with distant metastasis and underwent resection of the primary tumor. Within the whole cohort, 16.2% (42/260) patients underwent palliative operation and 5.8% (15/260) patients received no operations or systematic therapy. The median follow-up time was 48.4±1.6 months and 51.0±1.8 months in training and validation cohort. The median time for relapse was 36.7±1.5 months and 35.0±1.6 months in training and validation cohort. The 1-, 3-, 5-year OS rates were 98.1%, 67.3%, 57.2% in training cohort and 97.1%, 74.8%, 62.7% in validation cohort. Other characteristics were described in Table 1 and no significant differences were detected between training and validation cohorts.

### Survival analysis and identification of risk factors in G-NENs

Risk factors for overall survival and relapse free survival identified from univariate analysis in training cohort were shown in Table 2. Kaplan-Meier analysis suggested that age (HR=1.042, 95%CI, 1.021-1.064, p<0.001), Ki67 (HR=1.030, 95%CI, 1.022-1.038, p<0.001), Mitoses (HR=1.013, 95%CI, 1.009-1.018, p<0.001), NLR (HR=6.327, 95%CI, 3.676-10.890, p<0.001), CEA/CA19-9 (HR=4.829, 95%CI, 2.837-8.218, p<0.001), T stage (HR=1.865, 95%CI, 1.101-3.161, p=0.021), distant metastasis (HR=6.260, 95%CI, 3.405-11.509, p<0.001), tumor size (HR=1.676, 95%CI, 1.009-2.785, p=0.046) were significantly associated with poor overall survival in G-NENs. Age (HR=1.053, 95%CI, 1.025-1.082, p<0.001), Ki67 (HR=1.032, 95%CI, 1.022-1.042, p<0.001), Mitoses (HR=1.019, 95%CI, 1.014-1.025, p<0.001), NLR (HR=7.997, 95%CI, 3.98-16.069, p<0.001), CEA/CA19-9 (HR=7.061, 95%CI, 3.562-13.995, p<0.001) were significantly associated with poor relapse free survival in G-NENs.

In the multivariate survival analysis, age, Ki67, mitoses, NLR, CEA/CA19-9 and distant metastasis were identified as independent prognostic factors for OS in G-NENs. The details of multivariate analysis of OS and RFS were listed in Table 2.

### Construction and validation of the nomogram

**Figure 2:**
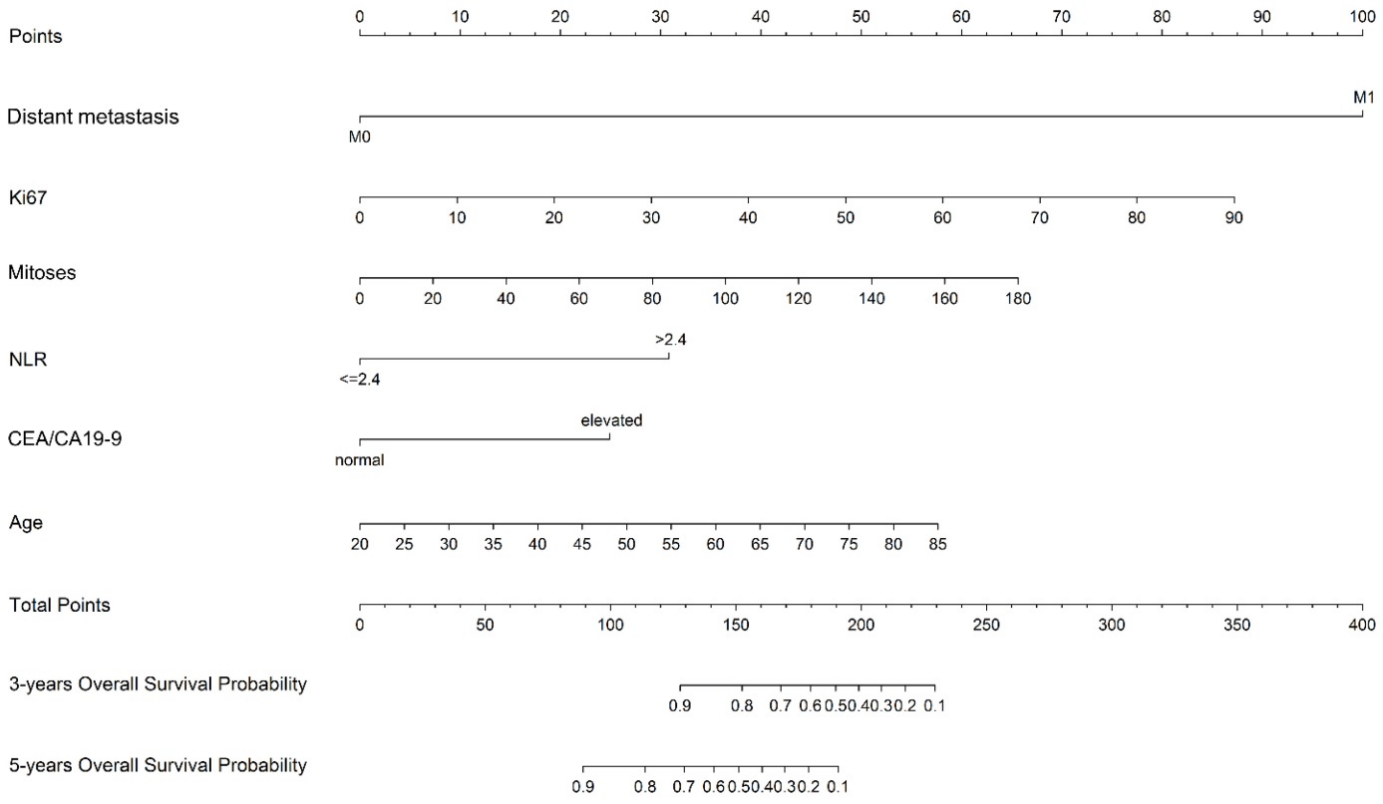
Nomogram predicting the 3-year and 5-year overall survival in G-NENs

The prognostic nomogram was constructed in training cohort based on multivariate analysis including six independent prognostic factors: age, Ki67, mitoses, NLR, CEA/CA19-9 and distant metastasis (Figure 2).

**Figure 3:**
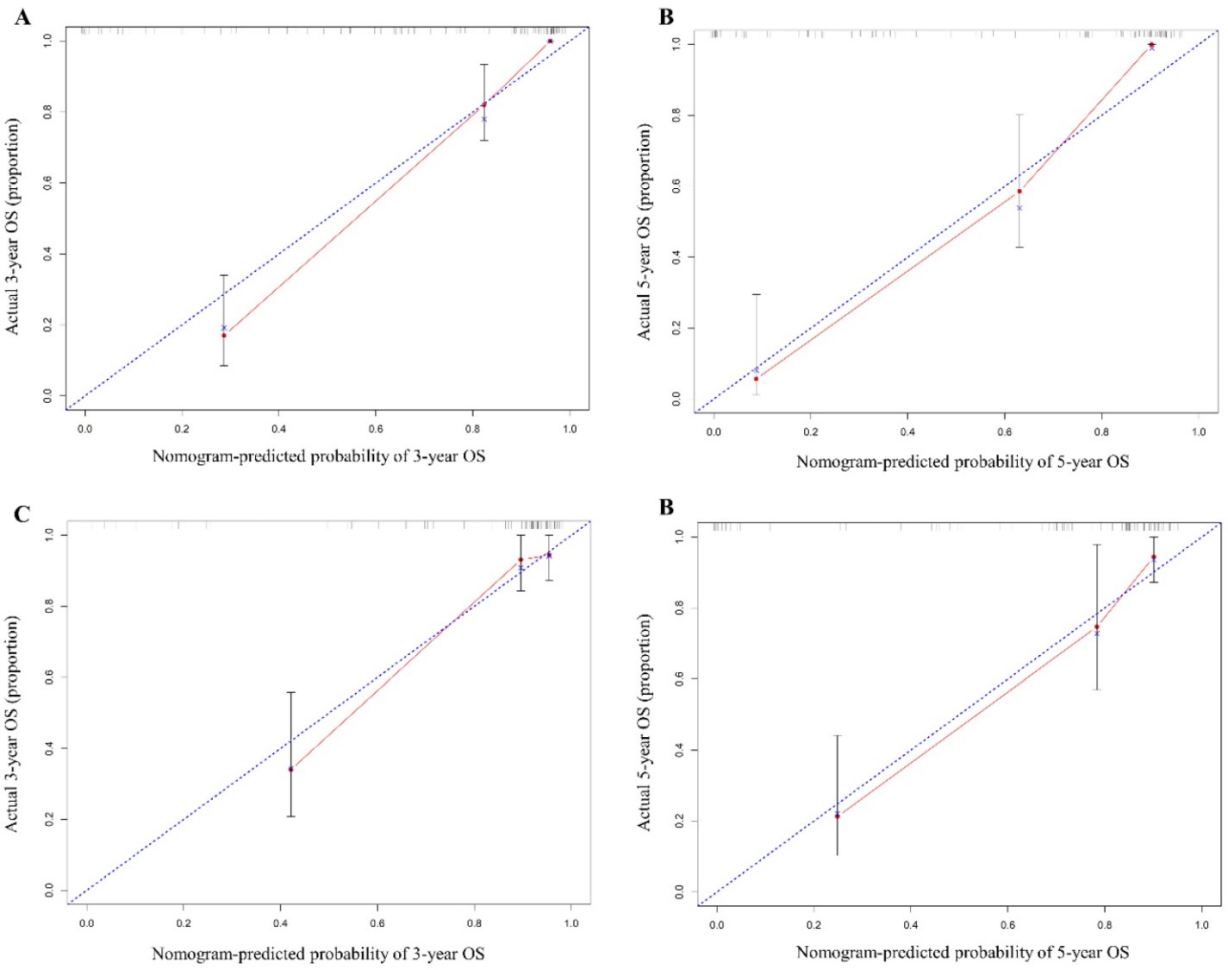
The calibration curve of nomogram in training (A & B) and validation cohorts (C & D). The calibration curve for predicting OS at 3 years (A for training cohort; C for validation cohort) and 5 years (B for training cohort; D for validation cohort) in G-NEN patients. The x axis represents the nomogram predicted survival rate and the y axis indicates the actual survival rate.

In the training cohort, our nomogram demonstrated a good ability to discriminate patients with good and poor prognoses with a C-index of 0.884 (95%CI, 0.846-0.922). As illustrated in Figure 3, the calibration plot showed a good agreement between actual observation and the nomogram prediction for probability of 1-year and 3-year overall survival.

To test the predictive value of the nomogram, we applied it in the validation cohort. The C-index of the nomogram was 0.852 (95%CI, 0.777-0.927). The calibration curve revealed consistency between actual and observed 1-year and 3-year overall survival(Figure 3).

### Comparison of nomogram with current predictive systems

We compared the predictive accuracy of our nomogram with both current G grading system and TNM staging system in G-NEN patients. In the training, the C-index values of WHO G grade system and AJCC TNM staging system were 0.760 (95%CI, 0.716-0.804) and 0.747 (95%CI, 0.689-0.723) respectively, which was significantly lower (P<0.001) than the C-index value of our nomogram. In validation cohort, the C-index of our nomogram is 0.852, which was also higher than the WHO G grade system (C-index=0.732, 95%CI, 0.660-0.804) and AJCC TNM staging system (C-index=0.811, 95%CI, 0.752-0.870). These results demonstrated that our nomogram, compared with the current G grading and TNM staging system, exhibited an enhanced discrimination in evaluating the prognosis of G-NEN patients (Supplemental Figure S1).

In addition, we applied the nomogram published by Fang in our training and validation cohort. 22 The C-index of Fang's nomogram was 0.751 (95%CI, 0.694-0.808) and 0.778 (95%CI, 0.703-0.853) in training and validation cohort, respectively, which indicated that our nomogram (C-index: 0.884 and 0.852) showed superior predictive value to Fang's in this data set.

Furthermore, we examined the survival prediction ability using AUC model. As demonstrated in Supplemental Figure 2, the AUCs of our nomogram were 0.922 (95%CI, 0.880-0.964) and 0.911 (95%CI, 0.856-0.966) in training and validation cohort, respectively. While the AUCs of WHO G grade system, AJCC TNM staging system and Fang's nomogram were 0.811 (95%CI, 0.745-0.877), 0.734 (95%CI, 0.647-0.835) and 0.767 (95%CI, 0.693-0.842) in training cohort, 0.774 (95%CI, 0.686-0.863), 0.787 (95%CI, 0.697-0.877) and 0.795 (95%CI, 0.708-0.881) in validation cohort (Supplemental Figure 2). These results suggested that our nomogram exhibited better predictive ability than current available prognosis models. The performance results of predictive models are summarized in Supplemental Table 2.

### Risk stratification and clinical use of nomogram

**Figure 4:**
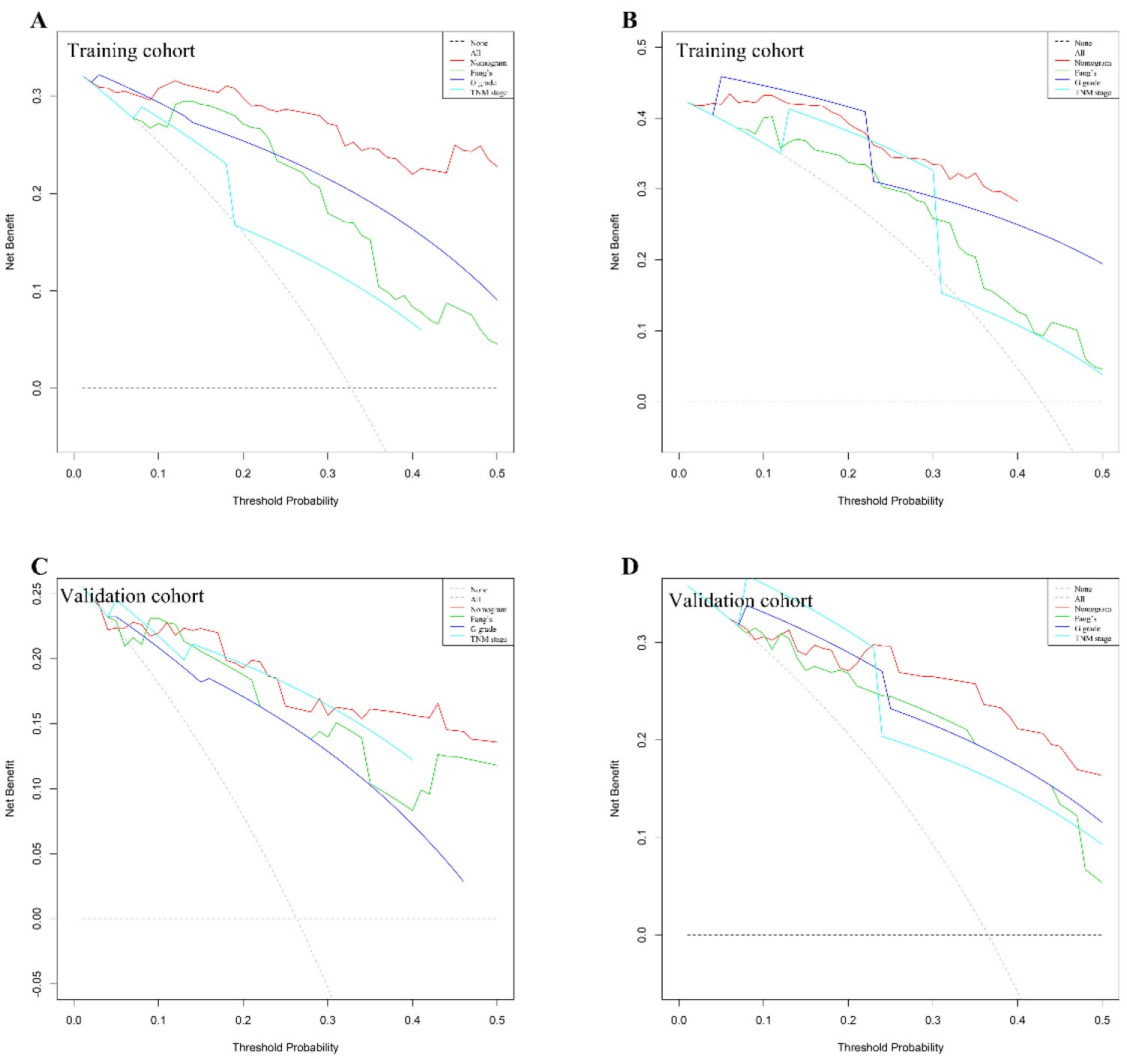
G-NENs decision curve analysis (DCA). Nomogram is compared with the G grade, AJCC TNM stage and Fang's nomogram, in terms of 3-year (A for training cohort; C for validation cohort) and 5-year (B for training cohort; D for validation cohort) overall survival.

We use DCAs to evaluate clinical usefulness of the constructed nomogram. Our results showed that the constructed nomogram has a better net benefit with higher threshold probabilities and improved performance for predicting 3-year and 5-year overall survival than G grade, TNM stage and Fang's nomogram both in training and validation cohorts (Figure 4). In training cohort, the mean risk score generated by the nomogram is 103±6. We categorized patients into 8 groups based on each individual score generated by the nomogram (Supplemental Table 3&4). To better distinguish and facilitate clinical use, we stratified patients into three distinct risk classes (low risk: <25th percentile, medium risk: 25-75th percentile, high risk: >75th percentile). In training cohort among 156 patients analyzed, 45 (28.8%), 51 (32.7%) and 60 (38.5%) patients were in low-risk, medium-risk and high-risk groups, respectively. As illustrated in Supplemental Figure 3A, the 5-year OS was 100.0%, 63.8% and 11.7% in low-, medium- and high-risk groups separately. Similarly, in validation cohort, 34 (32.7%), 40 (38.5%) and 30 (28.8%) patients were stratified into three risk group. The 5-year overall survival rate was 96.9%, 66.1% and 18.5% in three groups respectively (Supplemental Figure 3B).

## Discussion

In this study a novel and clinical applicable nomogram was developed to predict survival for patients with G-NENs. The multivariable Cox regression analysis identified 6 variables as independent prognostic factors and a nomogram was created based on these factors. These 6 factors--Age (HR 1.030), Ki-67 (HR 1.013), Mitoses (HR 1.011), NLR (HR 2.346), Serum CEA/CA19-9 (HR 2.013) and distant metastasis (HR 7.023) --could be accessed by clinical or pathological examinations which were applicable for clinician to evaluate individual outcome in G-NEN patients. We applied the nomogram in an external validation dataset and proved that the nomogram has a good predictive value (C-index for calibration is 0.852, 95%CI 0.777-0.927).

The recent increasing incidence of neuroendocrine tumors is related to the development of diagnostic techniques and the rising public awareness of the disease. 1 The large-scale Surveillance, Epidemiology, and End Results Program (SEER) study from United States in 2017 showed that the overall incidence rate for neuroendocrine tumors increased from 1.09 cases per 100,000 people per year in 1973 to 6.98 cases per 100,000 people per year in 2012. 2-3 The two most well accepted prognostic systems for G-NENs are TNM classification from AJCC and G grade system from WHO in 2010. 7,8 The former includes clinical parameters such as tumor characteristics (T), lymph node status (N), and distant metastasis (M), while the latter is based on pathological reports of mitoses count and Ki-67 index. In the present study, we developed a novel nomogram integrating more predictors accurately predict overall survival in G-NEN patients.

We compared the predictive ability of our nomogram with G grade and TNM staging system. The C-indices of our nomogram were 0.884 and 0.852 in training and validation cohorts, which demonstrated better discrimination values than G grade (C-index 0.760 for training and 0.732 for validation cohort) and TNM staging (C-index 0.747 for training and 0.811 for validation cohort). More recently *Fang et al* published a novel nomogram with larger sample size (n=1183) including five variables (age, tumor size, differentiation, lymph node and distant metastasis) to predict overall survival in GEP-NEN patients. 22 We validated Fang's nomogram with our two independent cohorts. Our results showed that C-indices of Fang's nomogram were 0.751 and 0.778 in training and validation cohorts respectively, which were lower than our predictive nomogram (0.884 and 0.852). Furthermore, we distinguished G-NENs from other NENs and included more variables, which achieved a superior specificity and performance of discrimination.

Based on multivariate analysis, we included six variables in our final predictive nomogram. According to WHO 2010 classification, the cut-off values of Ki-67 index to categorize G1, G2 and G3 are <3%, 3-20% and >20% respectively. 7 Various studies have showed that Ki-67 index and mitoses count are independent prognostic factors for GEP-NENs. 28-30 RGETNE study from Spain collected clinical information from 2813 GEP-NEN patients and found that 5-year OS rates for G1, G2 and G3 were 86%, 73% and 28% respectively. The multivariate analysis identified G3 (vs. G1/G2) as an independent prognostic factor (HR 2.333, 95%CI 1.668-3.264). 31 We applied Ki-67 and mitoses count as continuous variables which could more precisely predict the overall survival compare to categorical variables. Distant metastasis and age have also been reported as predictors for OS in GEP-NEN patients. 31-33 The latest population-based investigation employing SEER database demonstrated that age ≥61yrs (HR 1.85, 95%CI 1.75-1.96) and distant metastasis (HR 5.05, 95%CI 4.64-5.50) showed a high correlation with overall survival. 1 In our analysis, distant metastasis (M stage) was a strong prognostic factor (HR 7.023) and age also correlated to poor OS (HR 1.030) in G-NEN patients.

Moreover, we found that neutrophil to lymphocyte ratio (NLR) is an independent prognostic factor in G-NENs. The interactions between systemic inflammatory response and cancer development have been widely revealed in several studies. 34-35 Recent studies also proved that high NLR is a poor prognostic factor in gastric and pancreatic neuroendocrine tumors. 36-37 Our results showed that NLR is a predictor for overall survival in G-NENs and integrate it into the final nomogram. Another novel finding of our study is that we identified the elevation of serum tumor marker CEA/CA19-9 as a poor prognostic factor for G-NENs. Limited studies reported the predictive role of serum tumor marker in neuroendocrine tumors. *Chen et al* found the CEA and CA19-9 is elevated in 11.2% and 12.4% GEP-NENs patients. Elevated CEA or CA19-9 is a poor prognostic factor based on univariate analysis (P<0.001).^38^ Our results are in accordance with the previous study and demonstrated that elevation of serum tumor marker CEA or CA19-9 is an independent poor prognostic factor for gastric NENs (HR 2.013).

Based on our nomogram, we stratified G-NEN patients into three risk groups. Our results showed that 5-year overall survival rates were significantly different among risk groups (100.0%, 63.8% and 11.7% in training cohort and 96.9%, 66.1% and 18.5% in validation cohort). Based on the risk stratification, our nomogram may provide evidence for clinicians to predict individuals with poor prognosis and suggest an intense surveillance schedule for the patients in high risk group.

In our training cohort, 21.8% (34/156) patients are with metastasis. Within these patients, 73.5% (25/34) underwent palliative operation. (Supplemental figure 4). Literatures reported that certain patients with metastatic neuroendocrine tumors may be beneficial from palliative operation. 39-40 Therefore, we include patients with distant metastasis to widen the scope of the nomogram. The present study has several limitations that should be taken into consideration when we interpret and apply the nomogram. First, relatively small sample size might limit the scope of our investigation. Nevertheless, literatures reporting nomograms in predicting outcome in various type of neuroendocrine tumors includes similar number of patients in development of the predictive model. 41-43 Therefore, it should be a reasonable number of patients in the training cohort of the present study. Second, due to the retrospective nature of two cohorts, our nomogram might inevitably have selection bias. Our nomogram did not include some potential prognostic factors such as serum CgA since these data were incomplete in our two datasets. 44 Therefore, further multicenter prospective studies with larger sample size are needed to validate and confirm our nomogram.

Despite the limitations mentioned above, there are still many valuable implications of this nomogram. First, the present study for the first time evaluated the independent prognostic factors in G-NENs, constructed and validated a convenient predictive model for clinical use. Second, we proved that our nomogram, integrated 6 variables, showed a superior predictive capability to current evaluation models. In conclusion, this nomogram can help physicians to predict specific individual overall survival in G-NENs and select high risk patients for more intense surveillance.

## Supplementary Material

Supplementary figures and tables.

## Figures and Tables

**Table 1:** The demographic and clinical characteristics of patients in training and validation cohorts

Variable	Training cohort (n=156)	Validation cohort (n=104)	Total (n=260)	p value
Age, median (IQR), y	58±13.8	57±12.1	57±13.1	0.972
Gender Male Female	64.1% (100/156) 35.9% (56/156)	67.3% (70/104) 32.7% (34/104)	68.0% (170/260) 34.6% (90/260)	0.596
G grade G1 G2 G3	31.4% (49/156) 16.0% (14/156) 52.6% (48/156)	34.6% (36/104) 19.2% (20/104) 46.2% (48/104)	32.7% (85/260) 17.3% (45/260) 50.0% (130/260)	0.397
Ki-67 index (%) <3 3-20 >20	35.9% (56/156) 27.6% (43/156) 36.5% (57/156)	34.6% (38/104) 19.2% (25/104) 39.4% (41/104)	36.2% (94/260) 26.2% (68/260) 37.7% (98/260)	0.802
Mitoses (/10HPF) <2 2-20 >20	42.3% (66/156) 26.9% (42/156) 30.8% (48/156)	45.2% (47/104) 34.6% (36/104) 20.2% (21/104)	43.5% (113260) 30.0% (78/260) 26.5% (69/260)	0.137
T stage (pT) T1 T2 T3 T4	24.7% (37/150) 24.0% (36/150) 26.7% (40/150) 24.7% (37/150)	28.8% (30/104) 22.1% (23/104) 31.7% (33/104) 17.3% (18/104)	25.8% (67/254) 22.7% (59/254) 28.1% (73/254) 21.2% (55/254)	0.460
N stage (pN) N0 N1	68.6% (107/156) 31.4% (49/156)	64.4% (67/104) 35.6% (37/104)	66.9% (174/260) 33.1% (86/260)	0.484
Distant metastasis No Yes	78.2% (122/156) 21.8% (34/156)	88.5% (92/104) 11.5% (12/104)	82.3% (214/260) 17.7% (46/260)	0.034
Tumor size <2cm ≥2cm	59.0% (92/156) 41.0% (64/156)	62.5% (65/104) 37.5% (39/104)	60.4% (157/260) 39.6% (103/260)	0.597
Blood NLR ≤2.40 >2.40	67.9% (106/156) 32.1% (50/156)	64.4% (67/104) 35.6% (37/104)	66.5% (173/260) 33.5% (87/260)	0.555
Serum CEA/CA19-9 Normal Elevated	75.0 % (117/156) 25.0% (39/156)	78.8% (82/104) 21.2% (22/104)	76.5% (199/260) 23.5% (61/260)	0.473
Operation Curative Palliative (R1/R2) No operation	74.4% (116/156) 19.9% (31/156) 5.8% (9/156)	83.7% (87/104) 10.6% (11/104) 5.8 (6/104)	78.1% (203/260) 16.2% (42/260) 5.8% (15/260)	0.133
Status Alive Dead	61.5% (96/156) 38.5% (60/156)	63.5% (66/104) 36.5% (38/104)	62.3% (162/260) 37.7% (98/260)	0.754
Relapse free survival, months, median (95% CI)	36.7 (35.2-38.2) (n=116)	35.0 (33.4-36.6) (n=87)	36.0 (34.9-37.1) (n=203)	
Follow-up, months, median (95% CI)	48.4 (46.8-50.0)	51.0 (49.2-52.8)	49.6 (48.4-50.8)	0.409

*CI* confidence interval, *IQR* interquartile range

**Table 2:** Univariate and multivariate analysis for overall survival and relapse free survival

Variable	OS (n=156) HR (95% CI)	P value	RFS (n=116) HR (95% CI)	P value
Univariate				
Age (years)	1.042 (1.021-1.064)	<0.001	1.053 (1.025-1.082)	<0.001
Gender Male Female	1 (Ref) 0.618 (0.342-1.088)	0.195	0.723 (0.573, 0.873)	0.255
Ki-67 index (%)	1.030 (1.022-1.038)	<0.001	1.032 (1.022, 1.042)	<0.001
Mitoses (/10HPF)	1.013 (1.009-1.018)	<0.001	1.019 (1.014, 1.025)	<0.001
Blood NLR ≤2.40 >2.40	1 (Ref) 6.327 (3.676-10.890)	<0.001	1 (Ref) 7.997 (3.98, 16.069)	<0.001
T stage (pT) T1/T2 T3/T4	1 (Ref) 1.865 (1.101-3.161)	0.021	1 (Ref) 1.575 (0.814, 3.048)	0.177
N stage (pN) N0 N1	1 (Ref) 1.243 (0.712-2.138)	0.671	1 (Ref) 1.445 (1.021, 1.869)	0.467
Distant metastasis No Yes	1 (Ref) 6.260 (3.405-11.509)	<0.001	N/A	N/A
Tumor size <2cm ≥2cm	1 (Ref) 1.676 (1.009-2.785)	0.046	1 (Ref) 1.330 (0.685, 2.581)	0.399
Serum CEA/CA19-9 Normal Elevated	1 (Ref) 4.829 (2.837-8.218)	<0.001	1 (Ref) 7.061 (3.562, 13.995)	<0.001
**Multivariate** Age	1.030 (1.007-1.053)	0.009	1.032 (1.000-1.068)	0.056
Ki-67 index	1.013 (1.001-1.024)	0.027	1.036 (1.001-1.024)	0.027
Mitoses	1.011 (1.003-1.018)	0.004	1.015 (1.008-1.023)	<0.001
NLR	2.346 (1.245-4.422)	0.008	NS	NS
Serum CEA/CA19-9	2.013 (1.148-3.529)	0.015	NS	NS
Distant metastasis	7.023 (3.253-15.162)	<0.001	NS	NS

OS, overall survival; RFS, relapse free survival; NLR, neutrophil-to-lymphocyte ratio; N/A, not applicable, NS, not significant.
