# Supplementary material for: A novel and validated nomogram to predict overall survival for gastric neuroendocrine neoplasms
Source: J Cancer. 2019 Oct 15;10(24):5944–54. doi: 10.7150/jca.35785 (PMC6856574; doi:10.7150/jca.35785)
Supplement: Supplementary file 1 — Supplementary figures and tables. [file jcav10p5944s1.pdf]

Supplemental table 1 TRIPOD checklist for nomogram development and validation

**Section/Topic**			**Checklist Item**	**Page**
**Title and abstract**				
Title	1	D;V	Identify the study as developing and/or validating a multivariable prediction model, the target population, and the outcome to be predicted.	√
Abstract	2	D;V	Provide a summary of objectives, study design, setting, participants, sample size, predictors, outcome, statistical analysis, results, and conclusions.	√
**Introduction**				
Background and objectives	3a	D;V	Explain the medical context (including whether diagnostic or prognostic) and rationale for developing or validating the multivariable prediction model, including references to existing models.	√
	3b	D;V	Specify the objectives, including whether the study describes the development or validation of the model or both.	√
**Methods**				
Source of data	4a	D;V	Describe the study design or source of data (e.g., randomized trial, cohort, or registry data), separately for the development and validation data sets, if applicable.	√
	4b	D;V	Specify the key study dates, including start of accrual; end of accrual; and, if applicable, end of follow-up.	√
Participants	5a	D;V	Specify key elements of the study setting (e.g., primary care, secondary care, general population) including number and location of centers.	√
	5b	D;V	Describe eligibility criteria for participants.	√
	5c	D;V	Give details of treatments received, if relevant.	√
Outcome	6a	D;V	Clearly define the outcome that is predicted by the prediction model, including how and when assessed.	√
	6b	D;V	Report any actions to blind assessment of the outcome to be predicted.	**N/A**
Predictors	7a	D;V	Clearly define all predictors used in developing or validating the multivariable prediction model, including how and when they were measured.	√
	7b	D;V	Report any actions to blind assessment of predictors for the outcome and other predictors.	**N/A**
Sample size	8	D;V	Explain how the study size was arrived at.	**N/A**
Missing data	9	D;V	Describe how missing data were handled (e.g., complete-case analysis, single imputation, multiple imputation) with details of any imputation method.	√
Statistical analysis methods	[illegible]a	D	Describe how predictors were handled in the analyses.	√
	[illegible]b	D	Specify type of model, all model-building procedures (including any predictor selection), and method for internal validation.	√
	[illegible]c	V	For validation, describe how the predictions were calculated.	√
	[illegible]d	D;V	Specify all measures used to assess model performance and, if relevant, to compare multiple models.	√
	[illegible]e	V	Describe any model updating (e.g., recalibration) arising from the validation, if done.	**N/A**
Risk groups	11	D;V	Provide details on how risk groups were created, if done.	√
Development vs. validation	12	V	For validation, identify any differences from the development data in setting, eligibility criteria, outcome, and predictors.	√
**Results**				
Participants	[illegible]a	D;V	Describe the flow of participants through the study, including the number of participants with and without the outcome and, if applicable, a summary of the follow-up time. A diagram may be helpful.	√
	[illegible]b	D;V	Describe the characteristics of the participants (basic demographics, clinical features, available predictors), including the number of participants with missing data for predictors and outcome.	√
	[illegible]c	V	For validation, show a comparison with the development data of the distribution of important variables (demographics, predictors and outcome).	√
Model development	[illegible]a	D	Specify the number of participants and outcome events in each analysis.	√
	[illegible]b	D	If done, report the unadjusted association between each candidate predictor and outcome.	√
Model specification	[illegible]a	D	Present the full prediction model to allow predictions for individuals (i.e., all regression coefficients, and model intercept or baseline survival at a given time point).	√
	[illegible]b	D	Explain how to the use the prediction model.	√
Model performance	16	D;V	Report performance measures (with CIs) for the prediction model.	√
Model-updatin g	17	V	If done, report the results from any model updating (i.e., model specification, model performance).	√

**Discussion**				
Limitations	18	D;V	Discuss any limitations of the study (such as nonrepresentative sample, few events per predictor, missing data).	√
Interpretation	9a	V	For validation, discuss the results with reference to performance in the development data, and any other validation data.	√
	9b	D;V	Give an overall interpretation of the results, considering objectives, limitations, results from similar studies, and other relevant evidence.	√
Implications	20	D;V	Discuss the potential clinical use of the model and implications for future research.	√
**Other information**				
Supplementary information	21	D;V	Provide information about the availability of supplementary resources, such as study protocol, Web calculator, and data sets.	√
Funding	22	D;V	Give the source of funding and the role of the funders for the present study.	√

*Items relevant only to the development of a prediction model are denoted by D, items relating solely to a validation of a prediction model are denoted by V, and items relating to both are denoted D;V. We recommend using the TRIPOD Checklist in conjunction with the TRIPOD Explanation and Elaboration document.

Supplemental table 2 Performance of predictive models in training and validation cohort

**Predictive models**	**Training cohort**				**Validation cohort**			
	**C-index**	CI	AUC	**CI**	**C-index**	CI	AUC	**CI**
**Nomogram**	0.884	0.846-0.922	0.922	0.880-0.964	0.852	0.777-0.927	0. 911	0.856-0.966
**G grade**	0.760	0.716-0.804	0.811	0.745-0.877	0.732	0.660-0.804	0.774	0.686-0.863
**TNM stage**	0.747	0.689-0.723	0.734	0.647-0.835	0.811	0.752-0.870	0.787	0.697-0.877
**Fang's nomogram**	0.751	0.694-0.808	0.767	0.693-0.842	0.778	0.703-0.853	0.795	0.708-0.881

Supplemental table 3 Risk stratification based on nomogram risk score in training and validation cohort

**Risk score (percentile)**	**Training cohort**		**Validation cohort**	
	Survival rate	Number of participants	Survival rate	Number of participants
**0-20 (>5th percentile)**	100% (8/8)	8	100% (3/3)	3
**21-30 (5-10th percentile)**	100% (9/9)	9	92.9% (13/14)	14
**31-44 (10-25th percentile)**	100% (28/28)	28	100% (17/17)	17
**45-70 (25-50th percentile)**	96.4% (27/28)	28	93.8% (15/16)	16
**71-95 (50-60th percentile)**	56.3% (9/16)	16	57.9% (11/19)	19
**96-119 (60-75th percentile)**	14.3% (1/7)	7	40% (2/5)	5
**120-215 (75-90th percentile)**	22.7% (10/44)	44	23.8% (5/21)	21
**>215 (<90th percentile)**	25% (4/16)	16	0% (0/9)	9
**Total**	61.5% (96/156)	156	63.5% (66/106)	104

Supplemental table 4 Statistical analysis of risk group

**Risk group**		**Training cohort**	**Validation cohort**
		p value	Survival rate
**1**	**2**	<0.001	0.006
	**3**	<0.001	<0.001
**2**	**1**	<0.001	0.006
	**3**	<0.001	<0.001
**3**	**1**	<0.001	<0.001
	**2**	<0.001	<0.001

Note. Risk group 1 (0-25th percentile), Risk group 2 (25-75th percentile), Risk group 3 (>75th percentile); p value was calculated by Fisher's exact test.

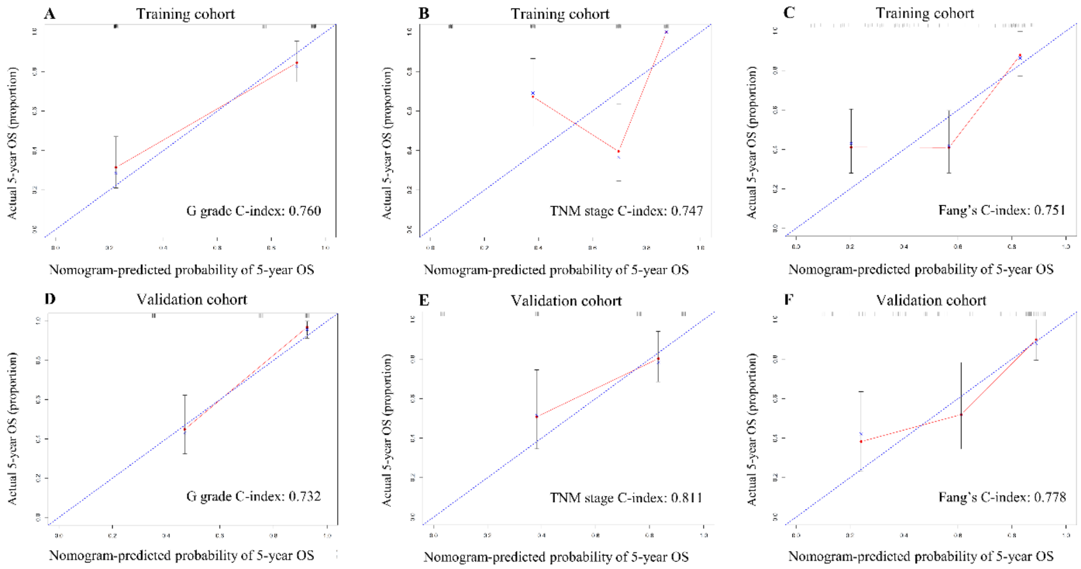

Supplemental figure 1. The calibration curve of different predictive models in training (A & B & C) and validation cohorts (C & D & E). The calibration curve for predicting OS at 5 year in training cohort (A for G grade; B for TNM stage and C for Fang's nomogram); The calibration curve for predicting OS at 5 year in validation cohort (D for G grade; E for TNM stage and F for Fang's nomogram).

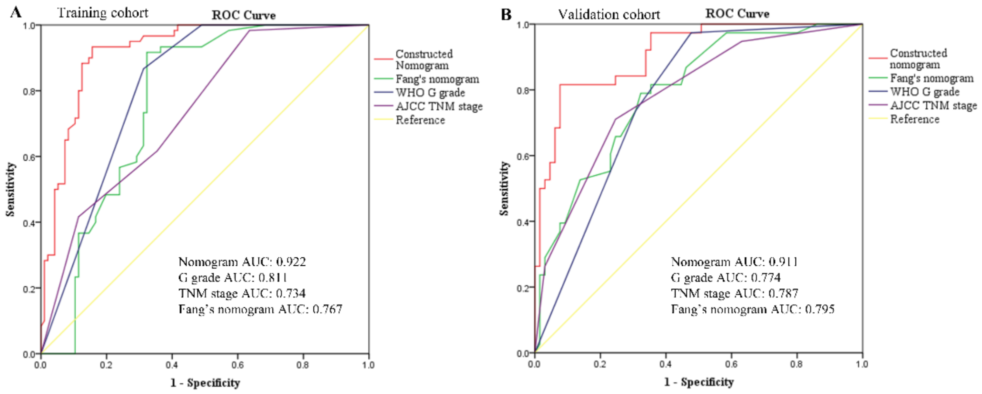

Supplemental Figure 2. Comparison of the AUCs of the nomogram and other predictive model in training **(A)** and validation **(B)** cohort.

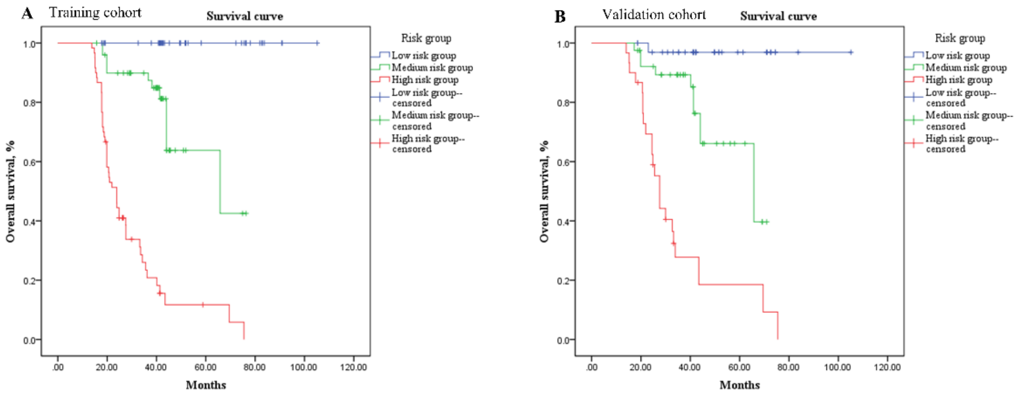

Supplemental Figure 3. Kaplan-Meier curves for overall survival in training **(A)** and validation cohorts **(B)** stratified by our constructed nomogram

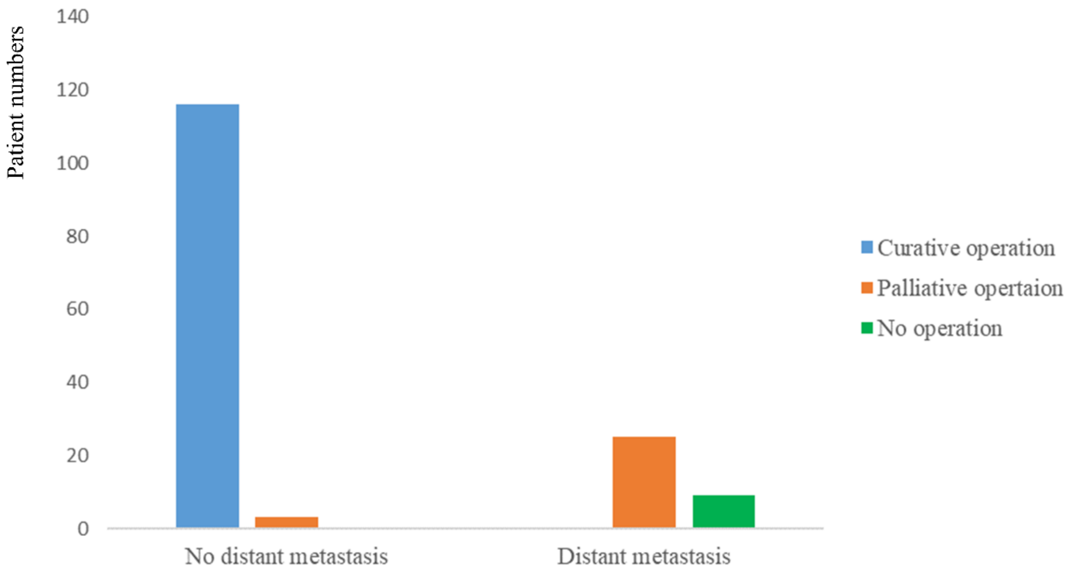

Supplemental figure 4. Operation types between patients with or without metastasis
